# Advances in Lasers for the Treatment of Stones—a Systematic Review

**DOI:** 10.1007/s11934-018-0807-y

**Published:** 2018-05-17

**Authors:** Peter Kronenberg, Bhaskar Somani

**Affiliations:** 1 Hospital Prof. Doutor Fernando Fonseca, Amadora, Portugal; 2 University Hospital Southampton NHS Trust, Southampton, UK

**Keywords:** Laser lithotripsy, Endourology, Pulsed thulium lasers

## Abstract

**Purpose of Review:**

Laser lithotripsy is increasingly used worldwide and is a continuously evolving field with new and extensive research being published every year.

**Recent Findings:**

Variable pulse length Ho:YAG lithotripters allow new lithotripsy parameters to be manipulated, and there is an effort to integrate new technologies into lithotripters. Pulsed thulium lasers seem to be a viable alternative to holmium lasers. The performance of similar laser fibers varies from manufacturer to manufacturer. Special laser fibers and “cleaving only” fiber tip preparation can be beneficial for the lithotripsy procedure. Different laser settings and the surgical technique employed can have significant impact on the success of laser lithotripsy. When safely done, complications of laser lithotripsy are rare and concern the endoscopic nature of procedure, not the technology itself, making laser lithotripsy one of the safest tools in urology.

**Summary:**

Laser lithotripsy has had several new developments and more insight has been gained in recent years with many more advances expected in the future.

## Introduction

This year is the 50th birthday when lasers first made their debut in urology [1], as well as the 30th birthday when urinary laser lithotripsy began establishing itself as a method of urinary stone treatment [2, 3]. Similar to most technological advancements, laser lithotripsy has also advanced with ongoing new developments. We therefore review the latest advances in laser lithotripsy to keep up to date with them.

## Material and Methods

A PubMed search was performed (December 2017) for papers including the terms “laser(s)” or “holmium” in association with any of the following terms “lithotripsy,” “lithiasis,” “stone(s),” “calculus,” “calculi,” “lithotripter(s),” “lithotrite(s),” “fiber(s),” “(endo)urology,” (endo)urologic(al),” or “intrarenal” in their title and published between the years 2015 and 2017, as well as 2018, to include already accepted, but not yet published papers. Additionally, the medical sections of ScienceDirect, Wiley, SpringerLink, and Mary Ann Liebert publishers where also searched for abstract presentations published in that time frame that were not indexed on PubMed. Moreover, key papers and other important studies on the subject were also included and cross-referenced, if they were considered noteworthy, despite being published before 2015. The authors adhered to PRISMA guidelines for this review [4]. All relevant data was identified, selected, and has been summarized below.

## Bibliographic Search Results

The PubMed search returned 1255 articles. Most of these articles (845) relate to basic laser research not necessarily related to medicine. They include research of new laser media and fiber production, soliton and quantum research, down to communications, nanotubes, and random bit generators created with lasers, all of them published in journals specialized in the wide-raging field of optics, and hence indexed in PubMed. Other 108 articles relate to the use of lasers in non-urological medical specialties such as ophthalmology, gastroenterology, ENT, vascular and general surgery, interventional radiology, and pneumology, as well as dermatology. The other 302 articles relate to urology-related fields, of which 150 concern the use of lasers in a non-lithotripsy-related setting, such as HoLEP, Greenlight laser, or other laser ablative techniques. Finally, the last 152 articles relate to urological laser lithotripsy. More than half of these papers are about case series, single-surgeon series, and comparisons of laser lithotripsy to other lithotripsy methods or case reports.

The search in the medical sections of ScienceDirect, Wiley, SpringerLink, and Mary Ann Liebert returned 12,454 papers or abstract presentations, 759 of them related to urinary stone treatments, some of them already picked up in the PubMed search. All abstract presentations of the major urology congresses were also reviewed and included [5–16].

The relevant data of the publications and abstracts have been categorized into the following four main groups: laser lithotripters, laser fibers, laser settings and technique, and laser safety and related complications (Table 1).

**Table 1:** Summary of the advances or technical aspects of laser lithotripsy, its complications, and their prevention

	Advancement or technical aspect	Benefit	Verdict
Laser lithotripters	Long pulse length (pulse duration or pulse width)	• Less fiber tip degradation • Less stone retropulsion • Smaller residual fragments • Ideal for “dusting”	Gradual rise in its use
	Moses effect (modulated laser pulse)	• More ablative (in vitro) • Less retropulsion	• No significant difference between lasing and procedural time in vivo • Limited availability (one manufacturer) • Costly
	Burst laser lithotripsy	• Greater ablation volume	• Likely to be used more often • Limited availability (one manufacturer)
	Thulium laser (pulsed)	• More ablative than Ho:YAG • Less retropulsion	• New technology • Very limited availability • Lack of clinical studies
Laser fibers	Ball tip fiber	• Easier insertion in deflected scope	• Initial benefit lost after a few seconds with degradation
	Tip cleaving tools	• All were equivalent	• Simple scissors are equally effective
	Leaving fibers coated	• Greater stone ablation • Easier to pass in the scope • Safer than stripped fiber	• More advantageous than stripped fibers in several categories
	Stripping of fibers	• Debatable higher stone ablation	• Significantly less advantages than coated fibers
Laser settings and technique	Fragmentation technique	• Faster ablation of primary stone	• Excellent for bladder or PCNL
	Dusting technique	• No fragments (dust) • No basketing • Decreased ureteral access sheath use	• Ablation itself takes more time, compensated by other time gains • Ultra-high-frequency lithotripters further shorten surgical time
	Pop-corning	• Ideal for multiple smaller stone fragments in an enclosed space Avoids endless chase of fragments	Helpful technique, complementing other lithotripsy methods
	Pop-dusting	• Similar to pop-corning, but creating more dust	• Helpful technique, complementing other lithotripsy methods
	*Complication*	*Prevention*	
Laser safety and related complications	Fever, subcapsular hematoma	• Reduce operative time • Use low-pressure ureterorenoscopy Confirm negative urine culture	
	Local thermal damage	• Never close irrigation • Intermittent laser use • Cooled irrigation if necessary	
	Eye damage	• Use eye glasses (simple ones will do) • Avoid laser fibers near eyes	
	Collateral instrument damage	• Keep fibers coated for better identification and regularly cleave them • Respect the safety distance between scope and laser fiber tip • Avoid passage of fibers through deflected scopes or else use BT fiber or at least a coated fiber	

### Laser Lithotripters

Laser technology was foreseen in 1917 and has been available and developed for 60 years [1, 17–19]. Most of the (initial) lasers emitted their laser energy in a continuous mode which has not been shown to be suited for lithotripsy [20], besides producing heat that can be harmful. On the contrary, pulsed lasers, e.g., the holmium:yttrium–aluminum–garnet (Ho:YAG) or the frequency-doubled double-pulse neodymium:YAG (FREDDY) laser [21, 22], deliver their energy in packets (pulses) that are suddenly released and very efficient at stone lithotripsy. Nevertheless, there are anecdotal reports of using Greenlight lasers for lithotripsy [23]. The reason for this difference in lithotripsy capabilities between continuous and pulsed lasers can be explained using the electric drill machine analogy: while trying to drill a hole in a wall, a continuous rotating drill is less efficient in perforating that wall and also generates much heat; on the contrary, a similar powered impact or percussion drill machine is much faster and efficient and also produces lower levels of heat. These features, together with its excellent safety profile, established and made the Ho:YAG laser ideal for laser lithotripsy in the last 30 years [2, 24, 25, 26••].

**Fig. 1:**
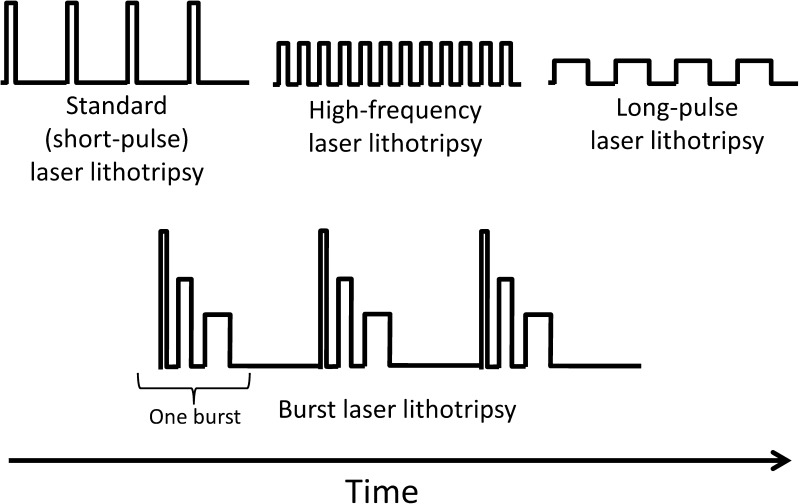
Graphic representation of differences between standard (short-pulse) laser lithotripsy, high-frequency laser lithotripsy, long-pulse laser lithotripsy, and burst laser lithotripsy over time

Despite the launch of more powerful and high-frequency Ho:YAG lithotripters over the years [27], they still only allowed the urologist to manipulate two parameters: pulse energy and pulse frequency. It took more than 20 years for holmium laser lithotripters to get their first, true technological upgrade, i.e., the ability to change pulse length, aka pulse duration or pulse width [26••]. Standard Ho:YAG lithotripters were restricted to short pulse lengths, while the newer lithotripters, besides short pulse, were now also capable of doing long-pulse lithotripsy (Fig. 1). Although all other lithotripter parameters (pulse energy, pulse frequency, and consequently total energy delivered) remain unchanged, in short-pulse mode, the energy delivered by a single laser pulse occurs during a short period of time (approximately 300 μs), while in long-pulse mode, that same amount of energy is distributed over a longer period of time (approximately 600 μs or more) [26••]. Pulse length was shown to be inversely correlated with ablation volume, i.e., the shorter the pulse length, the more ablative is the setting. Extreme pulse length comparisons of ultra-short and long-pulse mode (150 vs 800 μs) showed an average 60.6% higher ablation volume difference favoring ultra-short-pulse mode [28]. Despite this apparent lower efficiency, long-pulse mode showed to produce less fiber tip degradation and stone retropulsion [26••, 29, 30, 31•]. However, there is still some controversy in the subject, with some authors considering long pulse to be as ablative as short pulse [32•], or studies showing short-pulse lithotripters producing less retropulsion than more powerful long-pulse lithotripters [33]. There is also the general belief that long pulse produces smaller residual fragments and promotes a more “dusting” technique.

Regardless of the Ho:YAG lithotripsy domination, thulium laser technology, known for its usefulness in prostate ablation using continuous laser emission, and consequently considered unsuited for lithotripsy, has evolved and is now capable of pulsed laser emission. Although its usefulness in lithotripsy was demonstrated back in 2005 [34], only recently has it gained more attention. It has shown to be 2- to 4-fold faster without any significant heat increase and also producing three times less retropulsion, sometimes none at all, in comparison to Ho:YAG lithotripsy [35–37, 38•]. Pulsed thulium lithotripsy looks promising; however, true clinical studies are still lacking.

There is a new technology developed for a high-power 120-W Ho:YAG lithotripter, called the Moses effect. The theory is that this lithotripsy mode emits a modulated laser pulse whose first part divides the water between laser fiber tip and the stone, allowing the second part of the pulse to hit the stone unobstructed, more efficiently and with less retropulsion. However, on a closer look at the official high-speed videos, it reveals to be made of two separate laser pulses with a short time interval between them, rather than a single “modulated” laser pulse. Surgical opinions and in vitro experiments have indeed shown that this Moses effect reduced retropulsion [39, 40] and is significantly more ablative; however, in vivo experiments did not show any significant differences in terms of lasing and procedural times between regular and Moses lithotripsy techniques [40].

Recently, a novel Ho:YAG laser lithotripsy mode has been developed: burst laser lithotripsy (Fig. 1). Each burst consists of three individual laser pulses, the first one being the more energy intense while the last one the least energy intense, having successive increasing pulse lengths and which are emitted in a rapid succession after one another [41•, 42, 43]. The novel burst mode is significantly more ablative, achieving 60% higher ablation volumes than standard lithotripsy at similar power and energy settings [41•]. Possibly, the synchronized continuous variation of pulse energy and pulse length of high-frequency burst pulses, throughout the same lithotripter setting, might improve and speed up laser lithotripsy procedures in the future.

There have been also some efforts to incorporate useful auxiliary technologies into laser lithotripters. These include real-time stone/tissue differentiation using autofluorescence preventing the laser from firing against any structure other than the stone surface [44], or the attempt of in vivo analysis of urinary stone composition [45]. Some manufacturers are also trying to make carefully designed user interfaces, because most lithotripters are not as user friendly [46•].

### Laser Fibers

Besides the lithotripter, the laser fiber used also plays a vital role in the lithotripsy procedure. It is already known that larger laser fibers hinder the flow of irrigation fluid, limit the flexibility of instruments, and produce more retropulsion as well as larger stone fragments [26••, 47, 48•, 49, 50]; hence, the urologists prefer for small-diameter laser fibers, especially in flexible ureterorenoscopy. Additionally, there is emerging evidence about significant performance differences (e.g., on flexibility, degradation, ablation capabilities) among laser fiber manufacturers and no single fiber seems to be an ideal performer in every situation [51••, 52••] corroborating older studies on the subject [53•]. These performance differences should also be considered together with the manufacturers’ misinformation and mislabeling issues about laser fiber diameters, which were discussed not so long ago and these issues are far from being settled [48•, 54]. Even small-diameter changes can have a critical impact, because they indirectly affect accessibility, visibility, efficiency, total surgical time, and the lithotripsy procedure as a whole [48•]. On the other hand, pulsed thulium lasers are able to use smaller diameter laser fibers, than the ones used with the Ho:YAG lasers, representing a clear advantage and affecting favorably on the abovementioned aspects [55].

Lately, much attention has been given to ball-shaped tip (BT) laser fibers. BT laser fibers are particularly interesting because of their reduced insertion force in a completely deflected working channel without damaging it [56]. However, those features are lost as soon as laser emission occurs, because of the laser fiber tip degradation, the so called “burn-back” effect. Hence, most of these BT fibers should be only used once for a single, deflected working channel passage and at the very beginning of the surgery, because after several seconds of laser emission, they exhibit exactly the same characteristics as standard laser fibers [57]. Additionally, no better ablation properties where found on these special (and more costly) designed fiber tips in comparison to single-use standard fibers [56, 57]. Regarding reusable fibers, they show exactly the same tip morphology and degradation as new single-use standard fibers, as well as similar performances as long as they are not damaged along their length or at the connector. Although reusable fibers are more expensive than standard single-use fibers, they decrease fiber costs after the third and subsequent uses [57]. Yet other authors have shown that the use of single-use laser fibers can help decrease the overall cost of flexible ureterorenoscopy [58].

Still, laser fiber degradation, fiber fracture with smaller bend diameters or burn-back at its tip, affects every fiber, regardless of its shape or type (single-use or reusable fibers). It has been demonstrated that higher pulse energies, shorter pulse lengths, or harder stone material are more detrimental to laser fibers, in particular to their tips [26••, 29, 59•]. But there are also some conflicting results from other authors claiming that there is a trend for less fiber fracture with higher pulse energies [52••].

Related to these degradation issues is laser fiber tip preparation. This old, but still current practice of laser fiber preparation can be done before and during the procedure (to “renew” the fiber tip) and consists of stripping off the terminal portion of the plastic fiber coating and then cleaving several millimeters off the end of the glassy fiber components using special instruments (e.g., laser fiber stripper, ceramic scissors) [60–62, 63••]. One publication compared several cleaving methods, demonstrating that the “scribe pen” cleaving tool produced the highest average power output. However, these measurements were done without any laser emission, thus without considering any degradation issues over time [64]. Another paper analyzed the influence of stripping and cleaving methods of laser fibers on lithotripsy performance. The authors concluded that coated fibers outperformed stripped fibers; were not exposed to possible initial cladding damage caused by stripping; prevented damage caused by tip cleavage; and avoided cladding or other silica components to break off during lithotripsy as well as being more visible during the treatment. They also concluded that simple cleaving methods such as using a metallic surgical scissor were as good as more costly methods, as long as the fibers remain coated [63••]. Some of these results favoring coated fibers have been confirmed by other researchers [65]. Another advantage of keeping the fibers coated is that most of these fibers are able to pass through all angles of deflections in most scopes, while stripped fibers cannot without being harmful to the scopes’ working channel [66]. However, there is also evidence that initial advantages of certain cleaving methods over another level themselves out and the fibers become quite similar in performance in the first minutes of lithotripsy, because of equal, short-term fiber degradation [65, 67, 68•]. However, even over this topic, there is controversy among researchers, with some advocating against routine cleaving [67], others endorsing fiber tip preparation and renewal after 15 min or 10,000 J of laser emission, which is also important for reusable fibers [63••, 68•, 69], while still others claim that stripped fibers achieve greater stone ablation [65].

There are also other developments that can change the laser fiber as we know it. One of them is a miniaturized integrated thulium laser fiber and a stone basket. This device may minimize stone retropulsion, increase scope flexibility, allow higher saline irrigation rates through the working channel, reduce material degradation compared with separate fiber and basket manipulation, and reduce laser-induced nitinol wire damage [70]. The other is a more peculiar development: a fiber optic muzzle brake tip made of stainless steel to apply on thulium laser fibers. Similar to muzzle brakes used in rifles and artillery canons to reduce recoil and redirect propellant gases sideways, this laser fiber muzzle brake not only reduces stone retropulsion by 85%, but also provides minimal fiber degradation and an efficient stone ablation [71].

### Laser Settings and Technique

Most experts agree that fragmentation settings with higher pulse energies (> 0.5 J) and shorter pulse lengths have advantages, because they speed up the process of breaking up a large stone into smaller pieces. This can be speedy and useful in the bladder or in kidney stones with a large caliber percutaneous approach. However in ureterorenoscopy, this technique may also turn a single large problem into multiple, more time-consuming smaller problems. This is the reason why many urologists prefer to use a “dusting” technique [72–74]. Although dusting settings with low pulse energies (0.2–0.5 J), higher frequencies, and preferably longer pulse lengths ablate less stone material per unit time, it has several advantages: it decreases the use of ureteral access sheaths and therefore reduces potential ureteral trauma [75••, 76]; basket-associated complications are reduced because dust is naturally eliminated [73]; in the long run, it even reduces operative time by 20–40% by avoiding lengthy extraction procedures [73, 75••, 76]. Besides using low pulse energies for dusting, some authors also recommend keeping the laser fiber slightly away from the stone to “defocus” it and produce smaller fragments [75••]. With the recent arrival of high-powered high-frequency long-pulse Ho:YAG lithotripters, ultra-high pulse frequencies are available (up to 80 Hz), further speeding up the dusting lithotripsy procedure [77–79]. Despite all this evidence, according to an international survey, most urologists still use lithotripter settings around 10 Hz and 0.8 J [80]. However, it must also be acknowledged that if the dust does not evacuate spontaneously, stone-free rates in patients can be lower and this increases the risk of future stone-related events [75••, 81].

All the aforementioned settings and modalities relate to contact lithotripsy, which constitutes the first (and usually the only) stage of the lithotripsy procedure. However, when numerous smaller fragments result, which are still big enough to need treatment, but too time-consuming to chase individually, a second stage (completion) non-contact lithotripsy can be performed. The aim of non-contact lithotripsy is to pulverize these fragments and allow their spontaneous passage [82], preferably in a smaller and enclosed space such as a calix to increase the efficiency [83]. Two different techniques can be employed. One is the “pop-corn” technique, whose optimal settings have been confirmed by using a higher pulse energy (≈ 1.5 J), usually associated with a high-frequency (20–40 Hz), long-pulse mode, as well as a small-diameter laser fiber, and taking as much time as possible to produce clinically insignificant fragments [84•]. The other one is the “pop-dusting” technique, quite similar to the pop-corn technique but using a lower pulse energy (0.5 J), resulting in finer fragments without compromising fiber tip burn-back [82].

However, fragment size may not only be related to laser lithotripter settings, but also on the surgical technique employed, i.e., how the surgeon approaches the stone with the laser, i.e., “perforating,” “chipping,” “cutting into pieces” vs working uniformly and tangentially on the surface, by “dancing” or “painting” the stone with the laser and taking care not to break off large fragments from the main stone [26••, 75••, 85, 86]. Thus, even the best dusting setting, when used improperly, can produce large stone fragments.

Concerning settings and technique, it is the authors’ opinion that one should use lower pulse energy levels, with long pulse length (thus achieving smaller residual fragments and minimizing retropulsion, as well as reducing laser fiber degradation), and very high frequencies as technically possible to “go faster” and speed up the procedure. The laser fiber should be moved uniformly over the stone, without chipping or fragmenting the stone. The surgeon should be aware of the resulting fragment size, and if necessary adjust the settings, or even use a pop-corn technique to finish the procedure.

### Laser Safety and Related Complications

Ho:YAG laser lithotripsy is an efficient and safe technology for the treatment of urinary stones in almost any patient group, ranging from young children to adults and older patients, pregnant women to spinal cord injury patients, from solitary to allograft kidneys, or patients on certain medications and anticoagulants. Its safety profile has been largely demonstrated and still is in countless and recent safety-oriented studies [87•, 88•, 89, 90•, 91–98, 99•, 100, 101•]. Yet there are still some direct and indirect complications and safety concerns about laser lithotripsy, which have been recently researched.

The most frequent complication in laser lithotripsy is fever [102]. Renal backflow and infected urine fluid reabsorption can be one of the causes, and curiously retrograde intrarenal surgery (RIRS) has higher total fluid absorption than percutaneous nephrolithotomy (PCNL) procedures [103], which makes sense considering the enclosed space in RIRS. The presence of a preoperative stent, obstructive pyelonephritis, a positive preoperative bladder urine culture result, female gender, increased stone size, or lengthy operating time are significantly associated with postoperative fever and risk of sepsis [104, 105]. Spinal cord injury or patients with severe motor disabilities are in particular risk of sepsis (27%) after laser lithotripsy [106].

Bleeding is also a concern and several cases of renal subcapsular hematomas have been reported with Ho:YAG laser lithotripsy [102, 107, 108]. Yet it is questionable that these infections or bleeding complications were exclusively related to the Ho:YAG technology itself, because they are also known to occur in ureterorenoscopy without the use of laser lithotripsy [109]. On the other hand, one should bear in mind that there are at least three reported mortalities resulting from ureteral perforation and retroperitoneal bleeding using the Ho:YAG laser, although it is not specified if it was during a lithotripsy or endoureterotomy procedure [110]. In any case, the risk of subcapsular hematoma can be reduced by avoiding prolonged endoscopy and performing ureterorenoscopy under low pressure [111, 112], and the importance of always having a negative urine culture before any RIRS cannot be overemphasized.

Lately, attention has been given to local temperature rise at the site of laser lithotripsy, i.e., in the ureter or the kidney. Multiple authors and papers have confirmed that holmium laser emission in long bursts, even at lower power settings, does indeed rise fluid temperatures (up to 70 °C or more), particularly when irrigation is closed, potentially causing tissue injury [113•, 114•, 115•]. Since thulium laser has comparable absorption properties in water, it also shows similar temperature rises as the Ho:YAG laser [116, 117]. Considering that only 4.18 J of energy is needed to rise the temperature of 1 mL of water by 1 °C [118], and that holmium laser energy is highly absorbed by water, it comes as no surprise, that even with modest lithotripter settings providing dozens of Joules per second to a few milliliters of water enclosed in a very small space (e.g., a segment of the ureter or a renal calyx), the temperature is able to rise considerably and literally cook the surrounding tissues. Therefore, an endourologist should be aware of the risk of temperature rise during laser emission and implement a variety of techniques (higher irrigation flow rates, intermittent laser activation, and potentially cooled irrigation fluid) to control and mitigate thermal effects during laser lithotripsy and avoid unnecessary damage of the surrounding tissues [113•, 114•, 119].

In summary, considering the aforementioned issues, and with very few exceptions, patient-related safety and complications are probably less dependent on the laser technology itself, but dependent on multiple other factors relating to the procedure, e.g., the surgeon’s skill, the use of ureteral access sheaths, basketing instead of dusting, good intraoperative visibility, and operative time [63••, 73, 75••, 110].

But laser safety is not only about patient-related problems, but also concerns the safety of urologists and other staff in the operating room (OR). Eye injury is one of the main concerns when lasers are used; however, it was demonstrated that Ho:YAG lasers can only cause damage when all the following three conditions are met: high-energy laser settings, at very close distances (0–5 cm), and with no eye protection. Simple eyeglasses are equally effective in preventing laser damage as special laser safety glasses, and should an eye burn still occur, it would be restricted to the cornea [120••]. Not surprisingly, no eye injuries were reported so far with the use of Ho:YAG lasers [110]. Considering all reported adverse events with Ho:YAG lasers, if there is harm, it is to the patient or the surgeon (minor skin burns for the latter), but never to non-medical operators [110].

Radiation exposure for the patient as for the surgeon is a familiar problem in laser lithotripsy. Studies have confirmed the positive correlation between stone burden and radiation exposure during laser lithotripsy. Hence, clinicians should consider strategies to reduce the total radiation exposure, such as using pulsed instead of continuous fluoroscopy [121], although there are even defenders of using no fluoroscopy at all [122].

Material-related safety is also an issue, because laser energy can be harmful to other instruments, in particular to the delicate scopes used in endourology. To prevent accidental material damage, several strategies are recommended: to keep the laser fiber tip coated for better identification and to know its whereabouts [63••] and at a reasonable safety distance from the optical end of the scope (approximately one fourth of the endoscope field of view) [123••]; to regularly cleave the fiber tip to prevent back burns and retrograde laser emission [69]; to avoid passing a laser fiber through a deflected ureterorenoscope, and if inevitable, it is better to opt for an unused BT fiber or a recently cleaved and coated (not stripped) laser fiber [57, 124]; to avoid narrowed scope deflections with active laser fibers, and if inevitable, to change to smaller core fibers since they are less likely to fracture [59•]; and to avoid laser emission adjacent to auxiliary instrument components such as guidewires or basket tip parts since they are susceptible to breakages [125, 126].

## Conclusions

After 20 years of few technological developments, with the arrival of variable pulse length Ho:YAG lithotripters, new lithotripsy parameters can be manipulated to their advantage by the endourologist. There is an effort to integrate new lithotripsy modes and helpful technologies into lithotripters, including the use of pulsed thulium lasers instead of the Ho:YAG. Laser fibers from several manufactures perform significantly differently from one another and BT laser fibers have some short-lived advantages that can also be obtained with standard laser fibers. Laser fiber stripping is always detrimental for their performance and fiber cleavage can be done securely with simple metallic scissors. There are several settings for contact and non-contact laser lithotripsy, each with their own advantages and disadvantages, but the importance of the procedural movements of the laser fiber by the surgeon to deliver the laser energy to the stone should not be neglected. There are some reported complications with laser lithotripsy, but most of them are procedure-related and not with the laser technology itself. The remaining few safety issues can be prudently avoided, making laser lithotripters one of the safest instruments urologists can use in any patient group.
